# Individual differences in response to positive and negative stimuli: endocannabinoid-based insight on approach and avoidance behaviors

**DOI:** 10.3389/fnsys.2014.00238

**Published:** 2014-12-22

**Authors:** Daniela Laricchiuta, Laura Petrosini

**Affiliations:** ^1^IRCCS Fondazione Santa LuciaRome, Italy; ^2^Department of Dynamic and Clinical Psychology, Faculty of Medicine and Psychology, University “Sapienza” of RomeRome, Italy; ^3^Department of Psychology, Faculty of Medicine and Psychology, University “Sapienza” of RomeRome, Italy

**Keywords:** personality traits, endocannabinoid system, dopaminergic system, reward system, fear system, neuroimaging

## Abstract

Approach and avoidance behaviors—the primary responses to the environmental stimuli of danger, novelty and reward—are associated with the brain structures that mediate cognitive functionality, reward sensitivity and emotional expression. Individual differences in approach and avoidance behaviors are modulated by the functioning of amygdaloid-hypothalamic-striatal and striatal-cerebellar networks implicated in action and reaction to salient stimuli. The nodes of these networks are strongly interconnected and by acting on them the endocannabinoid and dopaminergic systems increase the intensity of appetitive or defensive motivation. This review analyzes the approach and avoidance behaviors in humans and rodents, addresses neurobiological and neurochemical aspects of these behaviors, and proposes a possible synaptic plasticity mechanism, related to endocannabinoid-dependent long-term potentiation (LTP) and depression that allows responding to salient positive and negative stimuli.

## Introduction

Many different labels have been proposed over the years to cover the definition of approach and avoidance. An Approach-Withdrawal distinction was introduced by Schneirla (1965) that argued that in all organisms the motivation is grounded in overt behavioral actions toward or away from stimuli. Subsequently, Davidson (1992) re-utilizing such a distinction presumed that action tendencies are grounded in differently lateralized cortical activation. In their analysis of emotion, Lang et al. (1997) used an Appetite-Aversion distinction to characterize two brain systems that underlie emotions: Appetite connotes consummatory and approach-oriented tendency, whereas Aversion connotes defensive and avoidance-oriented tendency. On the other hand, Lewin (1935), Miller (1944), and McClelland et al. (1953) conceptualized an Approach-Avoidance distinction in terms of valence-based processes, rather than over behavior. More recently, Elliot and Church (1997), Elliot and Thrash (2002), Elliot (2006), and Elliot (2008) addressed the issue, proffering the Approach-Avoidance distinction that expands the previous Approach-Withdrawal distinction in terms of energization of the behavior by (motivation), or direction of the action toward (behavior), positive stimuli in the case of the approach, and in parallel, energization of the behavior by, or direction of the action away from, negative stimuli in the case of the avoidance. Thus, positive or negative valence of the stimulus is considered the core of Approach-Avoidance distinction. The approach and avoidance behaviors appear to be the primary reactions to novel, rewarding, and dangerous stimuli on which all successive responses are based in order to gain successful adaptation. The approach system is considered a motivational system that activates reward-seeking behavior associated with impulsivity/exploration, whereas the avoidance system is considered an attentional system that promotes appetitive response inhibition or active overt withdrawal (McNaughton and Gray, 2000; Pickering and Gray, 2001; Carver and Miller, 2006).

The approach and avoidance behaviors are biologically based and constitutionally ingrained, since all organisms, following a phylogenetic gradient, are “preprogrammed” to approach or avoid particular classes of stimuli (Elliot, 1999, 2005, 2008; Elliot et al., 2006). The phylogenetically early mechanisms engender low-level responses to concrete stimuli, and complex mechanisms mediate sophisticated responses to a broader range of stimuli (Elliot et al., 2006). Approach and avoidance behaviors have been described not only across but also within phyla. Within the same species, some individuals have a greater tendency to approach or avoid a stimulus, also in relation to the age and context. For example, both in humans and animals, very young individuals are more sensitive than adults to the experiences linked to approach and avoidance, as early socialization or desensitization (Rothbart and Bates, 1998; Jones and Gosling, 2008; Sullivan et al., 2008). The adolescents exhibit emotional lability, impulsivity and proclivity to seek rewards and novel sensations (Fairbanks, 2001; Spear, 2002; Adriani and Laviola, 2004; Hefner and Holmes, 2007; Good and Radcliffe, 2011), even if sometimes these tendencies are maintained in adulthood (Roberts et al., 2001; Henderson and Wachs, 2007; Krishnan et al., 2007). However, increased sensitivity to reward is reversed in adolescents who are characterized in early childhood as having a behaviorally inhibited temperament (Helfinstein et al., 2011).

Excessive approach or avoidance behavior can lead to psychopathological disorders, as attention-deficit/hyperactivity disorders, depression and substance abuse on one hand, or anxiety and post-traumatic stress disorders on the other hand (Meyer et al., 1999; Muris et al., 2001; Kasch et al., 2002; Mitchell and Nelson-Gray, 2006). Thus, individual differences in approach and avoidance may represent predictors of vulnerability (or resilience) to neuropsychiatric diseases. Many of these conditions show sex differences in age of onset, risk, prevalence and symptomatology (Lynch et al., 2002; Costello et al., 2003; Rutter et al., 2003; Zahn-Waxler et al., 2008). In adolescence and adulthood, testosterone might increase susceptibility for some neuropsychiatric conditions by tipping the balance between approach and avoidance. For example, testosterone decreases avoidance by attenuating unconscious fear-responses (Hermans et al., 2006, 2007) and reducing sensitivity to punishment (van Honk et al., 2004), as well as it increases approach by enhancing sensation- and reward-seeking behaviors (van Honk et al., 2004; Coates and Herbert, 2008) and motivation to act (Campbell et al., 2010; Bos et al., 2012). The females exhibit a prolonged avoidance duration in a computer-based approach-avoidance task (Sheynin et al., 2014a,b). However, females may have a higher propensity for cocaine-induced approach-avoidance conflict (Back et al., 2005; Zakharova et al., 2009). In particular, the behavioral effects of drug rewarding stimuli vary across the reproductive cycle with specific “at risk” phases in respect to reward seeking. For example, women report higher drug-induced pleasure during the follicular phase than during the luteal phase (Evans et al., 2002), and female rats display greater reward-seeking behavior during estrus compared to other cycle phases (Feltenstein and See, 2007; Kerstetter et al., 2008, 2013).

## Conceptual space of approach and avoidance behaviors

Motivation is based on an intricate array of active approach and avoidance mechanisms. Functionally, approach and avoidance motivation are viewed as instigators of valenced propensities. They influence immediate affective, cognitive, and behavioral inclinations in response to real or imagined stimuli and orient individuals consistently across domains and situations. In humans, although some actions may derive directly and invariably from these proclivities, the ultimate behavior may be self-regulated and subjected to strategic planning, so that individuals can override their initial inclinations and redirect behavior (e.g., putting an approach behavior into action to override a basic avoidance tendency). The separate systems for approaching incentives and avoiding threats show individual differences and are sustained by disparities in brain structure and function. Personality traits are linked to neurobiological measures, such as neurotransmitter metabolites (Cloninger, 1986, 1987; Limson et al., 1991; Cloninger et al., 1993; Kim et al., 2002), markers that are associated with *in vivo* neuroimaging (Sugiura et al., 2000; Canli et al., 2001; Youn et al., 2002; Kumari et al., 2004), and morphometry (cortical thickness and volumes) in specific brain regions (Yamasue et al., 2008; Gardini et al., 2009; DeYoung et al., 2010; Picerni et al., 2013; Laricchiuta et al., 2014b,c,d). Approach and avoidance are related to and distinct from the central constructs of personality related in turn to the *trait adjective, affective disposition*, and *motivational system* constructs (Gable et al., 2003; Quilty and Oakman, 2004).

*Trait adjective* includes extraversion and neuroticism. Extraversion is the tendency to be sociable, active, optimistic, and to have high sensitivity to positive stimuli. Conversely, neuroticism is the tendency to be worrisome, prone, emotionally unstable, insecure, and to have high sensitivity to negative stimuli (Eysenck, 1981; Costa and McCrae, 1992). The specific sensitivity to positive or negative stimuli affects perceiving, attending, thinking, encoding, and recalling such stimuli. Eysenck (1981) proposed that extraversion is linked to a general cortical “arousability” and that neuroticism correlates with a low threshold for activation in the limbic system. In accordance, Eisenberger et al. (2005) suggested that neuroticism is the result of a neural system that detects a mismatch between actual and expected situations—a function that is carried out by the dorsal anterior cingulate cortex. DeYoung et al. (2010) reported that neuroticism covaries positively with the volume of the cingulate gyrus and negatively with the volume of the dorsomedial prefrontal cortex and posterior hippocampus—regions that are associated with threat, punishment, and negative affect. Recent results have shown that cerebellar white matter (WM) and gray matter (GM) volumes negatively covary with neurotic personality traits (Schutter et al., 2012). In parallel, extraversion covaries positively with the volume of the medial orbitofrontal cortex, which mediates the processing of reward-related information (DeYoung et al., 2010). Further, a positive association between patterns of synchronous neuronal activity and extraversion has been described in the cerebellum (Wei et al., 2011).

*Affective disposition* includes positive and negative emotionality, i.e., the tendency to experience positive or negative emotion and engage life in a positive or negative manner, respectively (Tellegen, 1985; Digman, 1990). Whereas positive emotionality is related to approach motivation and is elicited by appetitive stimuli (hedonic stimuli, reward cues, safety signals), negative emotionality is associated with avoidance motivation and is elicited by aversive stimuli (negative stimuli, threat cues, punishment signals). Individuals with high positive emotionality exhibit high energy, optimism, and openness toward others and the future. They tend to focus on the pleasant characteristics of themselves and others. Individuals with high negative emotionality exhibit high levels of distress, anxiety, irritability, fear, pessimism about the future, and dissatisfaction. They call attention to their own unpleasant characteristics and those of others. Electroencephalographic recordings revealed that positive and negative emotionality is associated with left and right prefrontal cortex activation, respectively (Wheeler et al., 1993). The link between the extraversion/neuroticism and the positive/negative emotionality is often discussed with regard to emotional reactivity. Extraverts and neurotics respond to stimuli with more intense emotions than introverts and non-neurotics. High levels of approach behavior in extraverts often lead to affective benefits. Unlike negative emotionality, which promotes withdrawal behavior, positive emotionality spurs exploratory behavior. The broaden-and-build theory of positive affect by Fredrickson (2001, 2004) suggests that once a positive emotionality is experienced, one seeks to expand and continue the experience that encourages the subject to approach novel situations, ideas, and individuals that are related to the object of interest. The author hypothesizes the development of an *upward spiral* in which positive emotions and the broadened thinking they engender influence one another reciprocally, leading to appreciable increases in emotional well-being over time. Positive emotions may trigger these upward spirals by building resilience and influencing the ways that people cope with adversity. Complementarily, the author hypothesizes a *downward spiral* in which negative emotionality and the narrowed pessimistic thinking it engenders influence one another reciprocally, leading to ever-worsening mood, till depression.

*Motivational system* includes behavioral activation system (BAS) and behavioral inhibition system (BIS). The reinforcement sensitivity theory proposes that the BAS produces positive affect and facilitates approach behaviors in response to conditioned appetitive stimuli, whereas the BIS generates negative affect and facilitates avoidance behaviors in response to conditioned aversive stimuli, especially in novel situations (Gray, 1987; Gray and McNaughton, 2000; McNaughton and Corr, 2004, 2014). Recently, Simon et al. (2010) examined the relation between individual differences in reward sensitivity and neural processing during expectation and reception of a reward, by using functional magnetic resonance imaging (MRI) during a monetary incentive delay task. Subjects with a high BAS exhibited greater activation of the ventral striatum during receipt of the reward, and greater activation of the medial orbitofrontal cortex during receipt and omission of the reward, demonstrating that approaching or avoiding reward-related situations have a distinct relationship with neural processing of the reward. Further, even amygdala responses appear to be positively associated with BAS (Beaver et al., 2008). Resting-state functional MRI demonstrated that BIS correlates negatively with the cerebellum and positively with the frontal gyrus (Kunisato et al., 2011). Increased fetal testosterone (FT) predicted increased BAS by biasing caudate, putamen, and nucleus accumbens to be more responsive to positively compared with negatively valenced information (Lombardo et al., 2012). In contrast, FT was not predictive of BIS, suggesting that testosterone in humans may act as a fetal programing mechanism on the reward system and influence behavioral approach tendencies later in life.

Interestingly, human approach-avoidance behavior has been assessed mainly by self-report questionnaires (e.g., Eysenck, 1981; Costa and McCrae, 1992; Cloninger et al., 1993; Taylor and Sullman, 2009), which query the respondent about the type and frequency of behaviors, and assign a score on each answer. Recently, in a human study on approach and avoidance tendencies the individual differences have been assessed on the Sensitivity to Punishment and Sensitivity to Rewards Questionnaire split into four subscales: Punishment that measures avoidance tendencies related to BIS; Impulsivity/Fun-Seeking, Drive, and Reward Responsivity that measure approach tendencies related in turn to BAS (Lombardo et al., 2012). Furthermore, to more directly evaluate avoidance behaviors, in humans several studies have used mild electric shocks (Lovibond et al., 2008, 2013; Delgado et al., 2009), or aversive visual or auditory stimuli (Dymond et al., 2011) as the aversive events that could be avoided. To evaluate approach behaviors, most human studies have employed monetary incentive tasks allowing the analysis of responses occurring during both expectation and receipt of reward or during the omission of reward (Schlund and Cataldo, 2010; Simon et al., 2010). A number of other studies have used the presentation of primary reinforcers, as somatosensory, olfactory or more often pleasant taste stimuli (O’Doherty et al., 2000, 2002). Another line of human studies has considered computer-based tasks (Molet et al., 2006; Schlund et al., 2010; Sheynin et al., 2014a,b), some of which take the form of a videogame, in the idea that even though no negative (e.g., electric shock) or positive (e.g., pleasant taste or money incentive) stimulus is delivered, people are nonetheless motivated to avoid aversive events and to approach rewarding events within the game. In the same vein, recently in a human study on approach-avoidance conflict a computer game was used in which the collection of monetary tokens provided the approach motivation, while the possibility that a virtual predator might wake up and remove all tokens provided a potential threat, and thus the avoidance motivation (Bach et al., 2014).

## Approach- and avoidance-related personality traits and brain structural variations

Within theories of personality, another model directly related to approach and avoidance is that related to the primary basic personality temperament and character traits by Cloninger (Cloninger, 1987; Cloninger et al., 1993). In his temperament and character inventory (TCI), he described four temperamental traits: Novelty Seeking (NS), Harm Avoidance (HA), Reward Dependence (RD), and Persistence (P). Novelty seeking is an approach-related personality trait and refers to the tendency to act. High NS scores reflect a greater tendency toward exploratory activity in response to novelty, impulsive decision-making, extravagant approaches to reward cues, and rapid loss of temper. The advantages of high NS are excitability, curiosity, enthusiasm, and quick engagement with anything that is new and unfamiliar. Conversely, its disadvantages are indifference, lack of reflection and intolerance to monotony, anger, inconsistency in relationships, and quick disengagement whenever a wish is frustrated. Harm avoidance is an avoidance-related personality trait and is the tendency to inhibit behaviors, acting with caution and apprehension. High HA scores indicate proclivity to respond intensively to aversive stimuli or signals of punishment or non-rewards, and they lead to pessimistic worry in anticipation of problems, fear of uncertainty, shyness with strangers, and rapid fatigability. The adaptive advantages of high HA are cautiousness and careful planning when a hazard is likely. Its disadvantages arise when a hazard is unlikely but still anticipated which leads to maladaptive inhibition and anxiety. Reward dependence is the inclination to maintain ongoing behaviors that have been associated with reinforcement and to express persistence, social attachment, and dependance on approval by others. High RD scores reflect to be tenderhearted, sensitive, dedicated, dependent, and sociable. The adaptive advantage of high RD is sensitivity to social cues, which facilitates affectionate social relations and genuine care for others. Its disadvantages are related to suggestibility and loss of objectivity, which are frequently encountered with people who are excessively socially dependent. Persistence refers to the ability to maintain arousal and motivation internally in the absence of an immediate external reward. High P scores indicate hard-working, perseverance, ambitiousness, and perception of frustration as a personal challenge. The adaptive advantage of a high P is the use of behavioral strategies when a reward is intermittent but the contingencies remain stable. Its disadvantages are related to perfectionist perseverance when contingencies change rapidly.

Within the factors that contribute to individual differences, gender influences HA (females have higher HA scores than males), and age influences NS (young subjects have higher NS scores than elders) (Cloninger et al., 1993; Fresán et al., 2011; Westlye et al., 2011). Although individuals with depression (Ono et al., 2002), bipolar mania (Loftus et al., 2008), schizophrenia (Fresán et al., 2007), substance use disorders (Conway et al., 2003), pathological gambling (Martinotti et al., 2006), and anxiety disorders (Kashdan and Hofmann, 2008) have NS or HA scores higher than healthy subjects, NS and HA are clearly non-dysfunctional behaviors and contribute to adaptive functioning. Further, NS and HA provide mechanisms to expand the range of stimuli and possibilities, protect one from potentially aversive contexts, supply the appropriate feedback for sculpting the brain and develop interest in specific domains. Structural neuroimaging studies on the regional specificity of brain-temperament relationships have demonstrated that the strength of fiber tracts from the hippocampus and amygdala to the striatum predicts the individual differences in NS (Cohen et al., 2009). Further, NS correlates positively with the volume of the frontal and posterior cingulate cortex; HA is negatively associated with the volume of the orbitofrontal, occipital, and parietal areas; RD correlates negatively with the volume of the caudate nucleus and frontal gyrus; P has a positive association with the volume of the precuneus, paracentral lobule, and parahippocampal gyrus (Gardini et al., 2009). Negative relationships between HA and anxiety-related traits and volumes of the entire brain (Knutson et al., 2001) and orbitofrontal (DeYoung et al., 2010) and left anterior prefrontal (Yamasue et al., 2008) cortices have been also reported. In parallel, increased HA is linked to decreased micro-structural integrity in widely distributed fiber tracts that include the corticolimbic pathways (Westlye et al., 2011). Furthermore, subjects with low NS and high HA scores have a relatively low striatal dopaminergic receptor density (Montag et al., 2010).

Assuming that the variability in an approach-related personality trait, such as NS, and an avoidance-related personality trait, such as HA, is normally distributed, in a large cohort of healthy subjects of both sexes and a wide age range (18–67 years), we tested the hypothesis that macro- and micro-structural variations in specific brain areas correlated with scores on the TCI temperamental scales (Picerni et al., 2013; Laricchiuta et al., 2014c,d). Region of interest (ROI)-based and voxel-based morphometry (VBM) analyses were used to assess macro-structural organization, and diffusion tensor imaging (DTI) scan protocol was used to evaluate micro-structural organization (Picerni et al., 2013; Laricchiuta et al., 2014b,c,d). Diffusion tensor imaging measures the diffusion of water molecules through tissues, detects micro-structural variations in the brain, and provides physiological information that is not available using conventional MRI (Le Bihan, 2007; Basser and Pierpaoli, 2011). The DTI indices that we used were Mean Diffusivity (MD) for GM and Fractional Anisotropy (FA) for WM, which reflect with great accuracy in space and time the subtle changes in cell structure which accompany various physiological and pathological states. In particular, low values in MD or high values in FA indicate high integrity and efficiency, and advanced organization of brain micro-structure. Variations in water diffusion parameters are linked to variations in cognitive functions (Piras et al., 2010, 2011) and personality dimensions (Westlye et al., 2011; Bjørnebekk et al., 2012, 2013).

**Figure 1:**
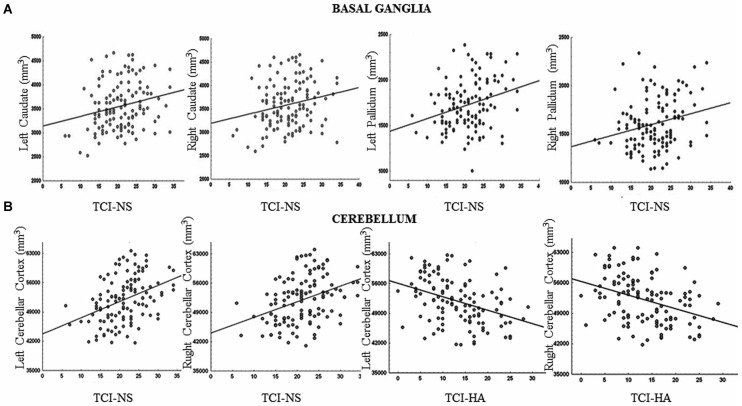
**Relationship between basal ganglia and cerebellar volumes and TCI scores. (A)** The volumes of the bilateral caudate and pallidum were positively associated with Novelty Seeking (NS) scores. **(B)** The volumes of the cerebellar cortex were positively associated with NS scores and negatively with Harm Avoidance (HA) scores. Scatterplots are separated for left and right volumes. Linear fits (solid black lines) are reported.

**Figure 2:**
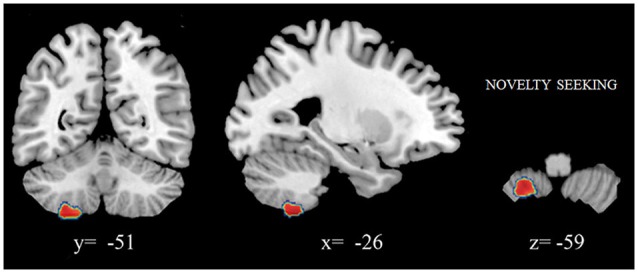
**Positive association between cerebellar gray matter volumes and NS scores**. Coordinates are in Montreal Neurological Institute (MNI) space. In figure left is left.

We found that increased volumes of the bilateral caudate and pallidum were associated with higher NS scores (Figure 1A), and increased MD measures in the bilateral putamen correlated with higher HA scores (Laricchiuta et al., 2014c). Further, greater cerebellar volumes were linked to higher NS scores, and reduced cerebellar volumes were associated with higher HA scores (Laricchiuta et al., 2014d; Figure 1B). These associations were observed in the cerebellar WM and cortex of both hemispheres. A greater-than-average volume might reflect greater-than-average power to perform specific functions. Human and animal evidence favors the larger-is-more-powerful position: training on particular tasks or experiencing complex environment increases the volume of functionally related brain structures (Boyke et al., 2008; Pangelinan et al., 2011; Di Paola et al., 2013). Thus, it is reasonable to assume that volume tends to covary positively with function. We also noted positive associations between the volumes of vermian lobules VIIb, VIII, and Crus 2 and NS scores (Figure 2; Picerni et al., 2013). The relationship between NS scores and cerebellar structures was also observed at the micro-structural level, as evidenced by the DTI data. The triad including increased volume, decreased MD, increased FA indicates that the macro- and micro-structural features of the posterior vermis support approach behaviors.

**Figure 3:**
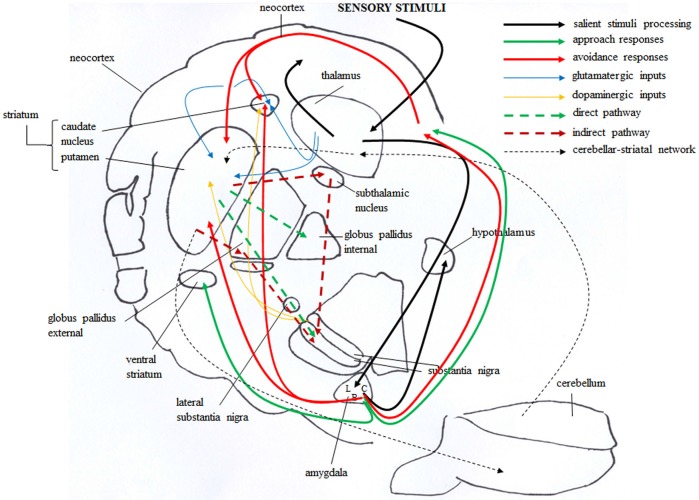
**Brain circuitries that mediate approach and avoidance behaviors**. Salient stimuli information from the sensory systems reaches the thalamus that in turn projects to neocortex and amygdala, first to its lateral (L) and then to central (C) and basal (B) nuclei (solid black line). The amygdala in turn projects to the hypothalamus, and directly or indirectly (via orbitofrontal cortex) to the dorsal striatum. These connections are involved in avoidance responses (solid red line). The outputs from the amygdala also reach the ventral striatum and the orbitofrontal cortex, and these connections are involved in approach responses (solid green line). The dorsal striatum receives also glutamatergic inputs (solid blue line) from neocortical and thalamic areas and dopaminergic inputs (solid yellow line) from the substantia nigra. These inputs establish synapses with striatal GABAergic cells, distinct in “direct” (dashed green line) and “indirect” (dashed red line) pathway projection neurons. Direct pathway projects to the internal globus pallidus and substantia nigra, whereas indirect pathway projects to the substantia nigra by way of the external globus pallidus and subthalamic nucleus. Also the bidirectional striatal-cerebellar network (dashed black line) is involved in the emotional and motivational processes linked to approach and avoidance.

These novel data that implicate a cerebellar substrate for approach- and avoidance-related personality traits extend the relationship between brain areas and personality to a structure that, until now, was believed to be involved primarily in motor and cognitive functions (Oliveri et al., 2007; Torriero et al., 2007; De Bartolo et al., 2009; Foti et al., 2010; Cutuli et al., 2011; Hampe et al., 2013), much less in emotional processes (Schmahmann and Sherman, 1998; Schmahmann et al., 2007; Timmann and Daum, 2007) and even less in personality individual differences (O’Gorman et al., 2006). Anatomo-clinical analyses indicate that the cerebellum is a critical neuromodulator of intellect and mood and that the posterior vermis, the so-called limbic cerebellum, chiefly regulates emotion and affect (Schmahmann, 2004; Stoodley and Schmahmann, 2010; Stoodley et al., 2012). Impaired executive and spatial functions, language deficits, and personality changes have been described in subjects with lesions of the posterior lobe and vermis (cerebellar cognitive-affective syndrome) (Schmahmann and Sherman, 1998). MRI studies have shown structural and functional abnormalities in the cerebellum in patients with personality, anxiety, or depression disorders (Pillay et al., 1997; De Bellis and Kuchibhatla, 2006; Fitzgerald et al., 2008; Baldaçara et al., 2011a,b). This evidence implicates the cerebellum in affective processing which affects personality characteristics. Moreover, the psychopathological profiles of patients who are affected by cerebellar diseases describe them as impulsive, obsessive, hyperactive, disinhibited, and developing ruminative and stereotypical behaviors—features that affect their personality style (Schmahmann et al., 2007). Even data in healthy subjects indicate limited capacity for emotional regulation after repetitive inhibitory transcranial magnetic stimulation over the cerebellum (Schutter and van Honk, 2009). The direct reciprocal connections between the cerebellum and basal ganglia (Figure 3, dashed black line) (Hoshi et al., 2005; Bostan and Strick, 2010; Bostan et al., 2010) constitute the neuroanatomical basis for the cerebellar influence on reward-related behaviors and motivation-related information processing—functions that, until now, have been attributed only to the basal ganglia (Wise, 2004; Delgado, 2007; Palmiter, 2008). It is likely that the cerebellum accelerates the “force” with which the reward is experienced (Schmahmann et al., 2007). Cerebellar activity signals when the sensory input differs from memory-driven expectations, provides a sensory prediction error, guides exploratory drive in novel environments, allows a flexible switching among multiple tasks or alternatives, and renders functions faster and more adaptive (Restuccia et al., 2007). The cerebellum performs these functions by refining the rate, rhythm, and force of the behavior and adjusting it for given situations. Essentially, the cerebellum receives information from the cortex and basal ganglia and sends a “corrected” signal back. In particular, based on cerebellar detection of error/novelty, Ito (2008) proposed that in the motor and cognitive domains the cerebellum develops both forward and inverse models. In the forward model, the cerebellum is informed by the cortex and basal ganglia with regard to information load, plans, and intentions about the upcoming behavior and on the characteristics of the environment in which the behavior is manifested. Thus, the cerebellum develops a progressive, short-cut, anticipatory model (Wymbs and Grafton, 2009; Seidler, 2010; van Schouwenburg et al., 2010). As the behavior and cognition are repeated and the anticipatory predicted feedback is received, the cerebellum becomes increasingly accurate in its predictive capacities and allows behavior to become faster, more precise, and independent of cortical control. With successful repetitions, behavior that is governed consciously by the cerebellar forward model becomes increasingly automated and the cerebellar “inverse” model is developed. This permits rapid and skilled behavior to occur at an unconscious level. The cerebellum is constantly constructing multipairs of models that constitute a complex modular architecture for adaptively regulating motor, cognitive, and emotional material. In triggering the new mental activity, the cerebellum could warn the prefrontal cortex about the absence of internal models that match the novel information, maintain the newly generated internal models, and incorporate them into routine schemes of thought. To successfully manage novelty, the cerebellum and neocortical/subcortical areas must be co-activated. Timing, prediction, and learning properties of the cerebellum, once integrated in the circuits that are formed with the neocortex, basal ganglia, and limbic system (Figure 3), could affect the control of complex novelty-related functions (D’Angelo and Casali, 2013). Thus, this widespread two-way communication sustains basal ganglia and cerebellar involvement in motor functions and cognitive and behavioral processing. Cortico-basal-cerebellar communication may influence and sustain even processes that are linked to individual differences in approach and avoidance behaviors (Figure 3, dashed black line). The basal ganglia and cerebellum have complementary roles in facilitating motivation that sustains and reinforces personality features. The positive correlation between basal ganglia and cerebellar volumes and NS scores and the negative association between basal ganglia and cerebellar volumes and HA scores are consistent with the varying levels of engagement that subjects with various personality traits require to their subcortical circuitries. In fact, subjects who search for unfamiliar situations, make the unknown known, explore new environments, display increased tendency toward risk-taking, sensation-seeking, and immediate reward-seeking, lack inhibition, as novelty seekers do, need very rapid detection of unfamiliar events, flexible switching among tasks, alternatives, and contexts, and fast adaptation to change. All these functions heavily engage basal ganglia and cerebellum.

## Approach and avoidance behaviors in animals

It is still very difficult to study the brain mechanisms of human subjective experience like emotion or motivation. Although the neuroimaging techniques are rapidly advancing, they reveal little about the precise working of neurons and trafficking of molecules in the brain activity related to approach and avoidance. Further, neuroimaging studies are correlative and cannot deliver answers about the nature and cause of the associations between structure and function. The techniques required to detail the mechanisms of brain functions usually cannot be used with humans for ethical and practical reasons, but animal research allows for use of these techniques, much as invasive they can be. In the following sections we address the experimental research on approach and avoidance behaviors, facing neurobiological, neurochemical and synaptic aspects.

### Tools for studying approach and avoidance behaviors

In a wide range of animal species individual differences in approach and avoidance behaviors have been observed, based on direction of the action toward positive (e.g., rewarding) stimuli or away from negative (e.g., dangerous) stimuli, on neophilic or neophobic responses, or on exploratory or withdrawal behaviors (Greenberg, 2003). In an attempt to model in rodents the human individual differences in approach and avoidance behaviors, many behavioral testing paradigms have been employed because almost all behavioral tests encompass approach or avoidance facets. In fact, although most tests are devoted to test spatial, discriminative, mnesic, attentive functions as well as emotional components, in many behavioral tests it is possible to emphasize the component of approach and avoidance. Overall, the tests integrate the approach-avoidance conflict designed to promote or inhibit an ongoing behavior characteristic for the animal, such as forcing or vise versa contrasting the tendency of mice to engage in exploratory activity, reward- or novelty-seeking behaviors, and social interaction. Notably, the explorative drive represents the prerequisite to recognize and seek for rewarding or novel stimuli and includes many components, such as suppression of the discomfort caused by unfamiliar spaces, exit from known starting areas, acquisition or use of efficient foraging strategies, and snapshots of the target view and representation-forming procedures.

Among the various tests, the mostly used are the Light-Dark Exploration Test, Social Interaction Test, Novelty-Induced hypophagia test, Approach-Avoidance conflict paradigm, Approach/Avoidance (A/A) Y-maze, and Open Field (OF) test (Bailey and Crawley, 2009).

As for the *Light-Dark Exploration Test*, the chamber is formed by a cage divided into two unequal compartments by a dark partition with a small aperture located in the bottom center. The smaller compartment is painted black and covered by a hinged lid. The larger compartment is uncovered with transparent sides and is brightly lit by fluorescent room lighting. Thus, the animal is exposed to environment with protected (dark compartment) and unprotected (light compartment) areas. The inherent conflict between exploratory drive and risk avoidance is thought to inhibit exploration. Most mice naturally demonstrate a preference for the dark protected compartment. The key measure for assessing approach-avoidance behavior is a willingness to explore the lighted unprotected area. Such proclivity is reflected in the number of transitions between compartments, and in the time spent in each compartment. An increase in exploratory activity is interpreted as a release of exploratory inhibition and novelty-seeking behavior. In fact, mice exhibiting higher levels of anxiogenic/avoiding-like behavior will make fewer transitions between the brightly illuminated, open area and the dark, enclosed compartment. Further, the time spent in risk assessment is another measure of anxiety/avoidance-related behavior. Risk assessment includes a stretch-attend posture in which the head and forepaws extend into the lighted area but the remainder of the body stays in the dark compartment (Bailey and Crawley, 2009).

As for the *Social Interaction Test*, unfamiliar animals are allowed to directly or indirectly interact in an arena. Time spent in interacting is recorded. Anxiolytic/approaching-like behavior is inferred if social interaction time increases and general motor activity remains unaffected. Conversely, decreased time spent in engaging social behavior indicates anxiogenic/avoiding-like behavior. The times engaged in aggressive (attack, aggressive unrest), avoiding (vigilant posture, escape and defense activity), approaching (following, social sniffing, over-under climbing) behaviors as well as in motor activities (rearing, walking) are scored (File and Seth, 2003).

*Novelty-Induced hypophagia test* is based on the typical behavior of the rodents that consume very limited quantities of any new even if highly palatable food and only after considerable investigation. This response is unconditioned, requires no training, and can be elicited in food-deprived or satiated animals by substituting a highly palatable food source for standard food. As the test sessions go on, the latency to the first taste decreases and the total amount of consumed food increases (Dulawa and Hen, 2005).

*Approach-Avoidance conflict paradigm* consists of a rectangular box subdivided into two compartments. One distinctive visual cue is associated with each compartment: one compartment has white walls and black floor, whereas the other one has black walls and white floor. For three consecutive days, the animal is placed in only one compartment that becomes familiar. In the following days, the animal placed in the familiar compartment is allowed to freely explore the whole apparatus (both familiar and novel compartments). The time spent in each compartment and frequency of crossings between compartments are indices of approach and avoidance behaviors (Adriani et al., 1998; Zoratto et al., 2013).

**Figure 4:**
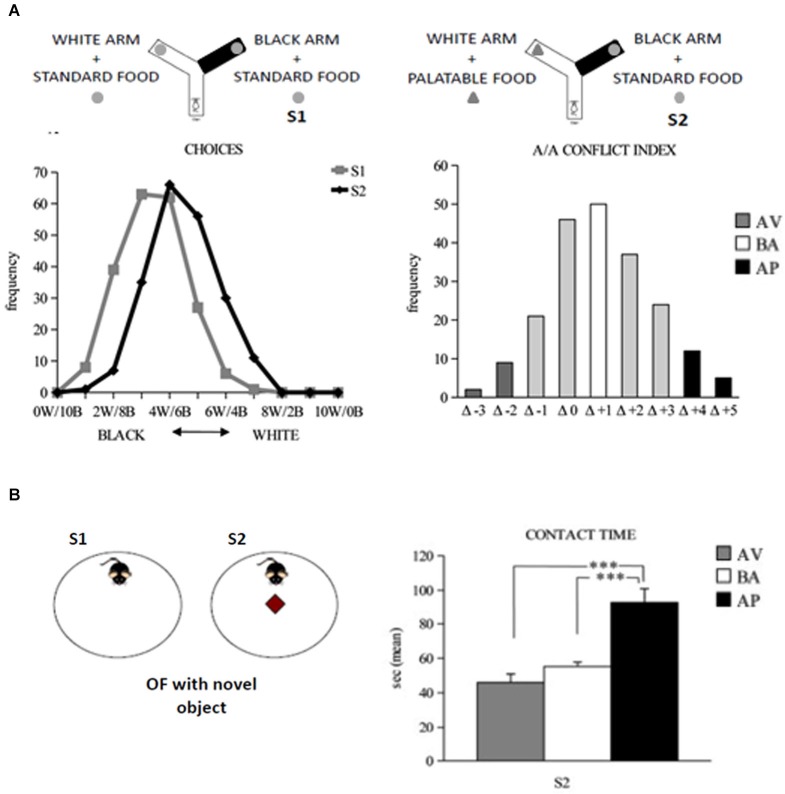
**Responses to conflicting stimuli of mice in A/A Y-Maze and OF task. (A)** Curves of distribution of the white and black choices of animals during the A/A Y-Maze sessions (on the left). Curve of distribution of the A/A conflict index, considered as the difference (Δ) in the number of white choices between sessions (on the right). **(B)** In the OF task (on the right), the AP mice significantly (*** *P* < 0.0005) spent more time in contacting the novel object than the AV and BA mice (on the right). Abbreviations: W: white arm; **B**: black arm; S1: first session; S2: second session; AV: avoiding animals; BA: balancing animals; AP: approaching animals. In **(B)**, data are presented as means ± SEM.

*A/A Y-maze* has a starting arm from which two arms stemmed, arranged at an angle of 90° to each other (Figure 4A). One of the two arms has black and opaque floor and walls and no light inside, while the other one has white floor and walls and is lighted. At the end of each arm of choice there is a food tray. The depth of the tray prevents mice from seeing the reward at a distance but allows for an easy reward (eating) and the appreciation of reward scent, not reducing the olfactory cues. Since the appetites for palatable foods have to be learned (Wise, 2006; Lafenêtre et al., 2009), a week before behavioral testing the animals have to be exposed to a novel palatable food (Fonzies, KP Snack Foods, Munchen, Germany) in their home cages for three consecutive days (Bassareo et al., 2002). At the beginning of behavioral testing, mice are subjected to 1-day habituation phase in which all Y-Maze arms are opened to encourage maze exploration. During habituation phase, no food is present in the apparatus. To increase the motivation to search for the reward, 12 h before exposure to the experimental set-up, the animals are slightly food deprived by limiting the food access to 12 h/day. Such a regimen has to result in no significant body weight loss. Testing phase consists of two 10-trial sessions with 1 min-inter-trial interval. In the Session 1 (S1), the mouse is placed in the starting arm and may choose to enter one of the two arms, both containing the same standard food reward. During the Session 2 (S2; starting 24 h after S1), the white arm is rewarded with the highly palatable food, while the black arm is rewarded with the standard food pellet. Thus, the A/A Y-maze task requires an animal to choose between two conflicting drives: reaching a new reward (highly palatable food) in an aversive (white and lighted) environment or reaching a familiar food (standard pellets) in a not aversive (black and opaque) environment. The considered parameters were: white choices, the frequency of entry into the white arm in S1 and S2; A/A conflict index, the difference in the number of white choices between S1 and S2; entry latencies exhibited in white and black arms, separately or regardless arm color or reward in each trial of both S1 and S2.

*Open field* apparatus consists of a wide circular arena delimited by a wall (Figure 4B). In S1, a mouse is allowed to explore the empty OF and its baseline level of activity is measured. In S2, the object is put in the arena center. Notably, the approach to the object requires the subject to overcome its innate fear toward open spaces and indicates thus that the animal is reacting to the mismatch between the initial (empty arena) and new (presence of the object) situations. Novelty preference is considered an inverse index of anxiety whereby an anxious mouse tends to avoid the potential dangers associated with a novel and unknown environment. The considered parameters were: total and peripheral distances traveled in the arena; central crossings; freezing duration; number of defecation boluses; latency and time of contact with the object.

In these tasks there is a clear conflict between positive and negative poles that simultaneously evoke approach and avoidance behaviors. Typically, when the positive and negative poles have similar strengths, the subject remains suspended or, at best, gravitates toward the slightly heavier pole of the conflicting situation. Many other tests are devoted to selectively assess behaviors of approach (as drug intake, response to positive conditioned stimulus, brain self-stimulation) or avoidance (as conditioned taste aversion, operant behavior to avoid an electric shock by a lever-press, aversive brain stimulation).

### Neurobiological aspects

Approach and avoidance behaviors are posited to emerge from mechanisms operative in the spinal cord (Berntson et al., 2003; Schutter et al., 2011), brain stem (Berridge and Peciña, 1995; Nelson and Panksepp, 1998; Challis et al., 2013) and cortex (Nasser and McNally, 2012). Namely, approach and avoidance behaviors are associated with the corticolimbic circuitry that comprises the prefrontal cortex, amygdala, and striatum and that controls cognitive functions, attention, reward sensitivity, and emotional expression (Figure 3; Cain and LeDoux, 2008; LeDoux, 2012; Bravo-Rivera et al., 2014). The intensity of appetitive or defensive motivation-related behaviors are modulated by the levels of neurotransmitters (dopamine, acetylcholine), neuropeptides (corticotrophin-releasing hormone, oxytocin, orexin), and neuromodulators (endocannabinoids) (Robbins and Everitt, 1996; Berridge, 2000; Gerra et al., 2000; Linfoot et al., 2009; Groppe et al., 2013; Mogi et al., 2014). Understanding neurochemical systems is crucial in addressing approach and avoidance topic (Tops et al., 2010). The avoidance situations (satiation, conditioned taste aversion, aversive brain stimulation) have the acetylcholine release in common, while the approach situations (eating, sugar bingeing, drug intake, positive conditioned stimulus, brain self-stimulation) have the dopamine release in common (Hoebel et al., 2007). However, it has to be considered that dopamine is an important factor also in responding to positive punishment provoked by the exposure to an aversive stimulus, and is involved in the motor aspects of both approach and avoidance behaviors. In the nucleus accumbens it has been demonstrated that dopamine and acetylcholine exert opposing roles in the control of GABAergic output in relation to approach and avoidance, and acetylcholine counteracts any excessive approach behavior mediated by the dopamine (Helm et al., 2003; Kelley et al., 2005; Hoebel et al., 2007). Interestingly, adult offspring of dams treated with corticosterone and a tryptophan-deficient diet showed increased avoidance behavior in the approach-avoidance conflict paradigm and anhedonia toward highly palatable reward in an operant progressive ratio test (Zoratto et al., 2013). These behaviors were associated with reduced dopamine and serotonin levels in the prefrontal cortex and reduced striatal and increased hypothalamic Brain Derived Neurotrophic Factor (BDNF) levels. Also neuropeptides are retained to be critical in approach and avoidance behaviors and have been much studied in animal research over the last several years. It has been demonstrated that in odor-recipient rats the odor cues from healthy conspecifics induced approach behavior, while the odor cues from sick conspecifics produced avoidance response (Arakawa et al., 2008, 2009, 2010a, 2011). In the odor-recipient rats, c-Fos mRNA expression was induced in olfactory bulb, amygdala, bed nucleus of stria terminalis, and hypothalamic paraventricular nucleus (Arakawa et al., 2010b). Interestingly, in the amygdala, the expression of oxytocin receptor mRNA was increased when the rats were exposed to healthy conspecific odor, while induction of arginine vasopressin receptor mRNA was found when exposed to sick conspecific odor. Into the amygdala the infusion of an antagonist of oxytocin receptor blocked approach behavior to “healthy” odor, while the infusion of antagonists of arginine vasopressin receptor inhibited avoidance response to “sick” odor. Thus, the approach and avoidance behaviors appear to involve similar brain regions but with different mechanisms (Ikemoto and Panksepp, 1999; Cain and LeDoux, 2008; Nasser and McNally, 2012). Recent findings indicate that also the orexins, hypothalamic neuropeptides that regulate feeding and sleeping behaviors, modulate avoidance behaviors. Rats treated with an antagonist of orexin-1 receptor approached a typically negative stimulus (cat odor) more than vehicle-treated rats (Staples and Cornish, 2014). Notably, exposure to cat odor induced Fos expression in the hypothalamus, suggesting that hypothalamic system is functionally involved with antipredator defensive behaviors (Blanchard et al., 2005). In accordance, microinjections of orexins in the paraventricular thalamic nucleus that innervates the amygdala decreased approach behavior to novelty in rats, indicating a negative emotional state (Li et al., 2010).

A very significant neuromodulatory system on approach and avoidance behaviors in humans (McDonald et al., 2003; Van Laere et al., 2009) as well as rodents (Pattij et al., 2007; Lafenêtre et al., 2009) is the endocannabinoid system (ECS) that deserves a detailed description.

As we recently demonstrated, spontaneous forms of approach and avoidance behaviors rely on ECS modulation in corticolimbic and striatal areas (Laricchiuta et al., 2012b, 2014a,d).

### Neurochemical aspects: endocannabinoid and dopaminergic systems

After their synthesis from arachidonic acid, endocannabinoids, such as anandamide (AEA) and 2-arachidonoylglycerol (2-AG), modulate synaptic transmission by stimulating cannabinoid type-1 (CB_1_) receptors (Freund et al., 2003; Piomelli, 2003; Marsicano and Lutz, 2006; Matias and Di Marzo, 2007; Kano et al., 2009). These receptors are primarily expressed in the corticolimbic, striatal and cerebellar pathways (Herkenham et al., 1990; Katona et al., 1999; Marsicano and Lutz, 1999; Palmiter, 2008; Koob and Volkow, 2010). Cannabinoid type-1 receptors presynaptically inhibit glutamatergic and GABAergic neurotransmission (Pagotto et al., 2006; Matias and Di Marzo, 2007; Kano et al., 2009) and this inhibitory control of excitatory and inhibitory neuronal subtypes determines the bimodal effects of endocannabinoids (Bellocchio et al., 2010). Thus, the ECS is engaged in myriad of physiological functions. During neural development, the ECS mediates neuronal proliferation, migration, and axonal growth (Berghuis et al., 2007; Harkany et al., 2008; Mulder et al., 2008; Trezza et al., 2008). Throughout life, the ECS influences synaptic transmission, neuroprotection, and neuroinflammation (Fowler and Jacobsson, 2002; Cota et al., 2003; Maldonado et al., 2006; Marsicano and Lutz, 2006; Kano et al., 2009; Lutz, 2009; Fowler et al., 2010). Further, the ECS governs emotional processes, anxiety, stress coping and extinction of aversive memories (Witkin et al., 2005; Lutz, 2007, 2009; Patel and Hillard, 2008; Laricchiuta et al., 2013). The involvement of the ECS in fear extinction is supported by the different responses of the human subjects genotyped for two polymorphisms of CB_1_ receptors in a fear-potentiated eyeblink startle reflex paradigm (Heitland et al., 2012). In adults with trauma-related psychopathologies, increased CB_1_ receptor availability in the amygdala is associated with increased attentional bias to threat and increased severity of the symptomatology linked to threat (re-experiencing, avoidance, and hyper-arousal), but not the symptomatology linked to loss (emotional numbing, depression, generalized anxiety) (Pietrzak et al., 2014). Also a common polymorphism that affects the enzymatic degradation of endocannabinoids by fatty acid amide hydrolase (FAAH) is linked to reactivity of the amygdala in relation to threat during a face allocation task involving fearful and angry faces, and to reactivity of the striatum in relation to reward in a gambling task with positive and negative feedback (Hariri, 2009). Further, the individuals with the FAAH polymorphism exhibit quick habituation of amygdala reactivity to threat (Gunduz-Cinar et al., 2013). Thus, the effects of the FAAH polymorphism demonstrate the engagement of ECS in the defensive and appetitive motivational systems (Conzelmann et al., 2012). Moreover, genetic deletion or inhibition of FAAH has context-dependent anxiolytic effects, as demonstrated in mice tested on Elevated Plus-Maze and Light-Dark Exploration Test (Naidu et al., 2007; Moreira et al., 2008).

In mice, experimental manipulations with strong rewarding and reinforcing properties, such as cocaine-induced conditioned place preference, spontaneous running wheel activity, and sucrose consumption, are associated with hypersensitivity of striatal GABAergic synapses to CB_1_ receptor stimulation (Centonze et al., 2007a,b; De Chiara et al., 2010). Conversely, social defeat chronic stress down-regulates CB_1_-controlled GABAergic striatal neurotransmission in mice (Rossi et al., 2008). Notably, the reinforcing effects of the primary rewards (food or drug) or the environmental stimuli associated with them enhance the dopaminergic release in corticolimbic and basal ganglia areas (Figure 3, yellow solid line) (Bassareo et al., 2002; Lupica and Riegel, 2005; Alcaro and Panksepp, 2011). Endocannabinoid system and dopaminergic system dynamically interact in controlling neuronal, endocrine, and metabolic responses to reward (Di Marzo et al., 2004; Fernández-Ruiz et al., 2010). In rats, the ECS inhibition on mesolimbic dopaminergic neurons influences the processes of attribution of salience to the reward represented by cocaine and heroin (De Vries et al., 2001; Fattore et al., 2003). The ECS has been implicated in several dopamine-related disorders, such as schizophrenia (Robson et al., 2014), Parkinson’s disease (Maccarrone et al., 2003), and drug addiction (Maldonado and Rodríguez de Fonseca, 2002; Rivera et al., 2013; Nader et al., 2014). In these conditions, ECS involvement likely reflects the activity of midbrain dopaminergic neurons and their target structures (Berke and Hyman, 2000; Everitt and Wolf, 2002; Castelli et al., 2011).

To analyze individual differences in spontaneous approach and avoidance behaviors, we tested adolescent (about post-natal day 32nd) C57BL/6JOlaHsd inbred mice in the A/A Y-maze (Laricchiuta et al., 2012b, 2014a,d). In the large sample of mice (more than seven hundred) tested in the A/A Y-maze task, we assigned the individuals into three phenotypes—avoiding (~6% of individuals that spontaneously reacted with withdrawing responses to the conflicting stimuli), balancing (~25% of individuals that reacted with balanced responses to the conflicting stimuli), and approaching (~7% of individuals that reacted with advancing responses to the conflicting stimuli, Laricchiuta et al., 2012b, 2014d; Figure 4A). All mice had similar explorativity levels in the initial trials of the task, but only approaching animals maintained high reactivity as trials went by. To eliminate the “food” and “palatability” dimensions and maintain the conflicting drives given by a new object placed in an anxiogenic central location of a wide arena, OF task has been used. In the OF, only the approaching animals were highly explorative and attracted by the new object (Figure 4B; Laricchiuta et al., 2012b). The close relation between approach behavior and explorativity has been proposed also in human studies that report that impulsivity and extraversion (Martin and Potts, 2004; Cohen et al., 2005), and risk aversion and low motivation (Tobler et al., 2007) are related to each other.

Because the A/A Y-maze and OF tasks integrate approach-avoidance conflict, the inevitable anxiogenic component that is linked to the conflict had to be considered. No differences in anxiety-related parameters of both tasks (defecation boluses, freezing times and central crossings) were found in the three phenotypes. Also, in the Elevated Plus-Maze, a well-validated anxiety test, all animals had similar anxiety levels.

To analyze the neuronal correlates of the approach and avoidance behaviors displayed by the three sub-populations of animals, we analyzed the CB_1_-mediated neurotransmission in medium spiny neurons (MSNs) of the dorsomedial striatum that is crucially involved in motivated and goal-directed behaviors (Palmiter, 2008; Koob and Volkow, 2010; Laricchiuta et al., 2012b). Presynaptic control of CB_1_ receptors on GABAergic transmission in the dorsostriatal MSNs was nearly absent in the avoiding animals but rose increased in the approaching animals. Specifically, application of a CB_1_ receptor agonist (HU210) to striatal slices provoked peak reductions of GABA_A_-mediated inhibitory postsynaptic currents of approximately 40%, 20%, and 0% in approaching, balancing, and avoiding animals, respectively. By enhancing the AEA endogenous tone with URB597, a drug that inhibits FAAH, the avoiding animals exhibited increased approach behavior and explorative drive. These behavioral responses were paralleled by the rescue of CB_1_ receptor sensitivity to HU210. On blocking CB_1_ receptors with AM251, a CB_1_ inverse agonist, the approaching animals reduced their contact times with object and explorative behavior in the OF task, behaviors accompanied by complete inhibition of CB_1_ receptor activity. Thus, the behavioral features of the avoiding and approaching animals treated with ECS agonists and antagonists tended to fade. In a nut shell, the treatment rendered them less inhibited and less “advanced”, respectively. These findings were confirmed by counterbalancing the pharmacological manipulations in avoiding and approaching animals. Avoiding animals that had a reduced CB_1_ control on GABAergic MSNs when further inhibited by AM251 treatment did not display any behavioral as well as electrophysiological modification in comparison to avoiding animals treated with vehicle. In parallel, approaching animals that had an enhanced CB_1_ control on GABAergic MSNs when further potentiated by URB597 treatment did not display any behavioral as well as electrophysiological modification in comparison to approaching animals treated with vehicle.

Balancing animals treated with URB597 developed a robust approach behavior toward palatable food in the A/A Y-maze and the new object in the OF task (Laricchiuta et al., 2014a). In these animals, the administration of AM251 alone or in combination with URB597 attenuated the approach behavior toward palatable food in the A/A Y-maze and the new object in the OF test, and suppressed the effects of HU210 on dorsostriatal GABAergic MSNs. These findings demonstrate that the effect of URB597 on approach behavior is mediated by CB_1_ receptors. Notably, in balancing animals, haloperidol (dopaminergic D_2_ receptor antagonist) blocked their approach behavior toward palatable food in the A/A Y-maze and the new object in the OF task, like AM251 did, and suppressed the effects of HU210 on dorsostriatal GABAergic MSNs (Laricchiuta et al., 2014a). These findings are consistent with the observation that D_2_ stimulation activates the dorsostriatal ECS, which in turn influences the GABAergic MSNs (Centonze et al., 2004, 2007a,b), and with the disparities in impulsivity that are associated with differences in monoamines in the striatum and nucleus accumbens in inbred rodents (Moreno et al., 2010).

In balancing animals, the co-administration of URB597 and haloperidol counteracted the effects of haloperidol on approach behavior in the A/A Y-maze but not in the OF task. Further, ECS potentiation combined with D_2_ receptor blockade arose only when the reward was represented by palatable food (Laricchiuta et al., 2014a). Such a facilitatory effect on food reinforcement was due to the higher salience of palatable food, based on the hedonic properties of its palatability, compared with the lower salience of the object, regardless of its novelty. On the electrophysiological level, CB_1_ receptor sensitivity to HU210 was rescued when URB597 and haloperidol were co-administered. These findings are consistent with the increased preference for palatable substances (evaluated by sucrose drinking) and sweet taste (evaluated by behavioral and electrophysiological responses to sweet mixtures) that is induced by the administration of exogenous cannabinoids or endocannabinoids (Higgs et al., 2003; Jarrett et al., 2005; Yoshida et al., 2010). In parallel, in rodents the AM251 treatment decreased the palatable food intake (Di Marzo and Matias, 2005; Pagotto et al., 2006). Further, mice injected with the selective CB_1_ antagonist Rimonabant repeatedly exposed to novel palatable food or a novel object, exhibited decreased reactivity to palatable food intake, but not to novel object (Lafenêtre et al., 2009). Cannabinoid type-1 antagonists decreased and CB_1_ agonists increased dopamine release induced by rewarding stimuli (Fadda et al., 2006; Solinas et al., 2006). Thus, by regulating the dopaminergic processes the striatal ECS increased the hedonic aspects of food-seeking, evaluated by an operant reinstatement procedure in rats (Duarte et al., 2004). Further, exogenous cannabinoids increased the hedonic reactions to highly palatable food (sucrose) but did not affect the reactions to aversive (quinine and saturated NaCl solutions) tastes. Consistent with the ability of cannabinoids to increase sucrose palatability, under cannabinoid pretreatment the sucrose induced a release of dopamine in the nucleus accumbens (De Luca et al., 2012).

As previously reported, enhanced or reduced CB_1_-mediated control on dorsostriatal GABAergic MSNs was associated with spontaneous approach/exploratory or avoidance behaviors, respectively (Laricchiuta et al., 2012b). A possible explanation for this observation could have been that approaching, balancing, and avoiding animals had varying densities of CB_1_ receptors and disparate activities of FAAH in the brain regions that govern the approach and avoidance behaviors. To test this hypothesis, we measured the density of CB_1_ receptors (by using [^3^H]CP55,940 binding autoradiography) and FAAH activity in many brain regions in the three subpopulations of mice (Laricchiuta et al., 2012a). Because significant changes in receptor density do not necessarily translate into gross alterations in receptor functionality or the presence of receptor reserve, we also examined CB_1_ receptor functionality (by using [^35^S]GTPγS binding autoradiography). Notably, only approaching animals had higher CB_1_ receptor functionality in the amygdaloid nuclei and hypothalamic dorsomedial nucleus. Interestingly, when compared with balancing animals, both approaching and avoiding animals, which attribute increased motivational salience to stimuli, had greater CB_1_ receptor densities in the amygdaloid nuclei and hypothalamic ventromedial nucleus. An intriguing parallel on the relation between opposite temperamental traits and similar receptor availability is provided by a PET study that reported the lower availability of striatal dopamine D_2/3_ receptors in healthy subjects with both high or low sensation-seeking, in comparison to subjects with moderate sensation-seeking (Gjedde et al., 2010).

Thus, the subcortical circuit that involves the amygdala and hypothalamus appears to drive individual differences in response to motivational cues, regardless of the opposite direction of the behavioral output. Amygdala mediates the processing of significant stimuli in conditioned fear learning (Pape and Pare, 2010), emotional memory (McGaugh, 2004; LaBar and Cabeza, 2006; LeDoux, 2012), assessment of novel (Schwartz et al., 2003; Weierich et al., 2010), ambiguous (Davis and Whalen, 2001), and threatening (LeDoux, 2000; Cain and LeDoux, 2008; Pape and Pare, 2010) stimuli. Further, in the amygdala, CB_1_ receptors presynaptically inhibit GABAergic neurotransmission (Freund et al., 2003). In theory, in avoiding and approaching animals the decreased inhibitory neurotransmission due to increased CB_1_ expression could influence the amygdaloid output that converges on other limbic regions, such as the hypothalamus that in turn mediates the reactive component (autonomic and somatic responses) of action. Hypothalamic ventromedial nucleus that regulates ingestive behavior and energy homeostasis exhibits the highest level of CB_1_ and cannabinoid receptor gene expression (Herkenham et al., 1990; Marsicano and Lutz, 1999; Jamshidi and Taylor, 2001; Pagotto et al., 2006). The increased CB_1_ density in the hypothalamic ventromedial nucleus in avoiding and approaching animals (and the greater CB_1_ functionality in the hypothalamic dorsomedial nucleus in approaching animals) could influence their autonomic and somatic responses and affect their phenotypes.

Overall, our data demonstrate that in response to conflicting stimuli, mice exhibit variance of spontaneous behaviors, ranging from avoiding to approaching (Laricchiuta et al., 2012b, 2014a,d). The increased hedonic response and explorative behavior of the approaching animals are linked to greater CB_1_-mediated control on dorsostriatal inhibitory neurotransmission. Conversely, the inhibitory response to reward of the avoiding animals correlates with decreased CB_1_-mediated control on dorsostriatal inhibitory neurotransmission. The robust differences among behavioral phenotypes in striatal CB_1_-mediated currents are not a direct consequence of striatal CB_1_ receptor expression levels, but they reflect more subtle changes in ECS signaling (Laricchiuta et al., 2012a). In this context, significant evidence indicates that striatal neurotransmission is important for generating anticipatory/preparatory responses in the presence of a conditioned stimulus paired with a positive or negative unconditioned stimulus (Berridge and Robinson, 1998; Ikemoto and Panksepp, 1999; Cardinal et al., 2002).

It has been proposed that the subjects that attribute higher salience to reward-related cues may be vulnerable to addiction (Flagel et al., 2009; Robinson and Flagel, 2009; Saunders and Robinson, 2010), and the subjects that show higher NS behavior may be vulnerable to depressive-like symptoms (Duclot and Kabbaj, 2013). Conversely, the subjects that attribute higher value to aversive cues may be vulnerable to anxiety and post-traumatic stress disorders (Bush et al., 2007; Yehuda and LeDoux, 2007). By using a Pavlovian conditioned approach procedure, Morrow et al. (2011) classified the rats based on whether they learned to approach and interact with a cue that predicted food reward (sign-tracker animals) or conversely learned to go to the location of the food delivery (goal-tracker animals). Sign-trackers were more fearful of discrete cues that predicted foot-shock, while goal-trackers exhibited greater contextual fear even in the absence of discrete cues, suggesting that a subset of individuals attributes high salience to predictive cues regardless of emotional valence. Because motivational systems have evolved primarily to support drives and to direct actions, their outputs facilitate information processing, motor recruitment, action readiness, and affective and attentional engagement.

## A possible synaptic scenario of approach and avoidance behaviors

As underlined by McNaughton and Corr (2014), the approach and avoidance behaviors have to be anchored to the long-term global sensitivities of the underpinning neural systems. Considering the huge bulk of experimental and human findings (see Elliot, 2008 for an overview), we propose a possible synaptic scenario of approach and avoidance behaviors.

**Figure 5:**
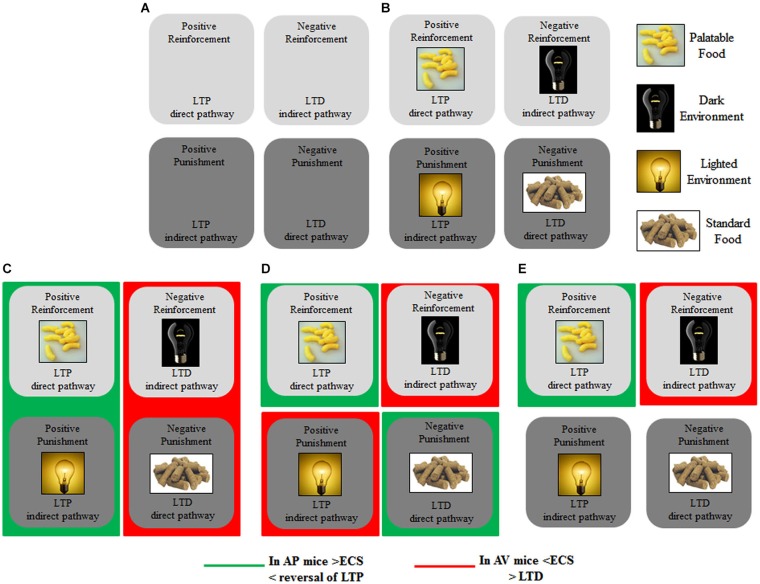
**Modeling striatal plasticity of direct and indirect pathways in reinforcement and punishment, related to approach and avoidance. (A)** Positive Reinforcement may be associated with LTP onto direct pathway neurons, whereas Positive Punishment may be associated with LTP of indirect pathway neurons. Negative Reinforcement may be associated with LTD onto indirect pathway neurons, whereas Negative Punishment may be associated with LTD of direct pathway neurons. **(B)** By applying this modeling to the A/A Y-maze task, the Positive Reinforcement is represented by Palatable Food; the Negative Reinforcement by Dark Environment; the Positive Punishment by Lighted Environment; the Negative Punishment by Standard Food. **(C)** ECS modulations of direct and indirect pathways may reduce the LTP reversal in the approaching animals, and increase the LTD in the avoiding animals. **(D)** By modulating the synaptic plasticity, ECS might shift the behavior toward the most significant component of a conflicting context (in the case of approach behavior: Positive Reinforcement against Negative Punishment; in the case of avoidance behavior: Negative Reinforcement against Positive Punishment). **(E)** By decreasing reversal of LTP, the potentiation of ECS of direct pathway may contribute to the approach behavior, prompting the animal toward the Positive Reinforcement; by increasing LTD, the de-potentiation of ECS of indirect pathway may contribute to the avoidance behavior, prompting the animal toward the Negative Reinforcement.

Figure 3 schematizes the main brain structures retained to mediate approach and avoidance behaviors. Information from the sensory systems reaches the thalamus that in turn projects to neocortex and amygdala, first to its lateral and then to its central nucleus (Figure 3, solid black line) (Pape and Pare, 2010). Outputs from the lateral to central and basal nuclei are critical in the increased processing of salient stimuli, whether they are pleasant or aversive (Cain and LeDoux, 2008). The amygdala in turn projects to the hypothalamus (Miguelez et al., 2001). Notably, in the amygdaloid and hypothalamic nuclei the avoiding and approaching animals display an increased density of CB_1_ receptors (Laricchiuta et al., 2012a). Furthermore, from the amygdala direct or indirect (via orbitofrontal cortex) outputs reach the dorsal striatum and these connections appear to be involved in avoidance responses (Figure 3, solid red line) (Lang and Bradley, 2008). The outputs from the basolateral and central amygdaloid nuclei reach the ventral striatum and the orbitofrontal cortex, and these connections appear to be likely contributors to the execution of approach behavior (Figure 3, solid green line) (Lang and Bradley, 2008). Since both amygdaloid-hypothalamic-striatal and striatal-cerebellar networks are involved in the emotional and motivational processes linked to putting into action behaviors toward or away from emotionally salient stimuli, the striatum that inherently serves as a gating mechanism represents a crucial crossroad in the neuroanatomical geography of approach and avoidance behaviors (McNab and Klingberg, 2008; Koziol et al., 2010). The goal-directed and hedonic nature of the striatal contribution to action is supported by pioneering studies on “compulsory approaching syndrome”, in which animals with striatal lesions compulsively followed and contacted humans, other animals, or even stationary objects (Villablanca et al., 1976), and on reinforcing and rewarding effects of striatal micro-stimulations in animals (Plotnik et al., 1972; Phillips et al., 1976, 1979) and humans (Lilly, 1960; Heath, 1963). The dopaminergic nature (Kilpatrick et al., 2000) of the reinforcing and rewarding effects has been conclusively confirmed by recent innovative optogenetic studies (Tsai et al., 2009; Bass et al., 2010; Adamantidis et al., 2011; Witten et al., 2011). Striatal neurons appear to not respond to movement *per se* but rather to features of the movement that supports reinforcement, such as the anticipation or expected reward value (Kawagoe et al., 1998; Schultz et al., 2000, 2003). However, striatal neurons and dopaminergic release play a role not only in reward processing but also in aversive processing (Ferreira et al., 2003, 2008; Pezze and Feldon, 2004; Matsumoto and Hikosaka, 2009; Bromberg-Martin et al., 2010; Cohen et al., 2012). Roitman et al. (2005) showed that distinct populations of striatal neurons respond to rewarding (sucrose) or aversive (quinine) taste. Besides the amygdaloid projections, the striatum receives also glutamatergic inputs from neocortical and thalamic areas (Figure 3, solid blue line) and dopaminergic inputs from the substantia nigra (Figure 3, solid yellow line). These inputs establish synapses with striatal GABAergic MSNs and cholinergic interneurons (Calabresi et al., 2014). The MSNs are distinct in “direct” and “indirect” pathway projection neurons (DeLong, 1990; Graybiel et al., 1994). Direct pathway MSNs project to the internal globus pallidus and substantia nigra pars reticulata (SNr; Figure 3, dashed green line), whereas indirect pathway MSNs project to the SNr by way of the external globus pallidus and subthalamic nucleus (Figure 3, dashed red line). The activation of the direct or indirect pathways facilitates or inhibits the motor output, respectively (Durieux et al., 2009). In this framework, Kravitz and Kreitzer (2012) propose that positive reinforcement (caused by the presence of a positive stimulus) may be associated with plasticity that enhances synaptic efficacy (long-term potentiation, LTP) onto direct pathway neurons, whereas positive punishment (caused by the presence of a negative stimulus) may be associated with LTP of indirect pathway neurons. Conversely, negative reinforcement (caused by the absence of a negative stimulus) may be associated with plasticity that depresses synaptic efficacy (long-term depression, LTD) onto indirect pathway neurons, whereas negative punishment (caused by the absence of a positive stimulus) may be associated with LTD of direct pathway neurons (Figure 5A). By applying this interesting schema to the approach-avoidance (A/A Y-Maze) task we used (Laricchiuta et al., 2012b, 2014a,d), the reinforcements and punishments can be labeled as depicted in Figure 5B. Notably, the substrate for the cross-talk between direct and indirect pathways is represented by ECS that induces the LTD of the dorso-striatal MSNs and of their afferent and efferent connections (Lovinger, 2010). However, an opposite synaptic consequence results when the activation of ECS is kept persistent. In fact, in the dorso-striatal MSNs the long-lasting activation of the ECS impairs both LTD and the reversal of LTP (Nazzaro et al., 2012), mechanisms of synaptic plasticity involved in the habit formation (as drug-related habits or compulsive behaviors) and in reinforcement- or reward-related behaviors (Gerdeman et al., 2003; Gerdeman and Lovinger, 2003; Kravitz et al., 2012; Nazzaro et al., 2012). Interestingly, in our approaching or avoiding mice the striatal ECS is potentiated or down-regulated, respectively (Laricchiuta et al., 2012b). It is reasonable to hypothesize, although it has been not yet demonstrated, that such ECS modulations may influence the mechanisms of synaptic plasticity, by reducing the LTP reversal in the approaching animals, and by increasing the LTD in the avoiding animals (Figure 5C). The next step of this chained modeling is linked to the rewarding or aversive nature of the direct and indirect pathways. Specifically, are the neurons activated by rewarding stimuli belonging to the direct pathway and the neurons activated by aversive stimuli belonging to indirect pathway? Optogenetic activation of direct or indirect pathway neurons heightens or impairs the strength of cocaine-induced conditioned place preference, respectively (Lobo et al., 2010). Consistently, the activation of direct or indirect pathway neurons heightens or impairs amphetamine sensitization (Ferguson et al., 2011). Furthermore, impaired dopamine-mediated transmission of direct pathway neurons reduces cocaine-locomotor sensitization and impairs conditioned place preference for a food reward, and conversely the impaired transmission of indirect pathway neurons evokes aversive learning deficits (Hikida et al., 2010). Moreover, the stimulation of direct or indirect pathway evokes the rapid learning to contact or to avoid a trigger, respectively (Kravitz et al., 2012), exerting then an opposite control over not just movement, as classically indicated (DeLong, 1990; Graybiel et al., 1994), but also on approach and avoidance behaviors. Thus, in response to the previous questions, it appears that the direct pathway activation is rewarding and indirect pathway activation is aversive. Once more it is possible to hypothesize that by modulating the synaptic plasticity of direct and indirect pathways neurons, the ECS might shift the behavior toward the most significant component of any conflicting context (in the case of approaching behavior: positive reinforcement against negative punishment; in the case of avoiding behavior: negative reinforcement against positive punishment), determining thus the ultimate behavioral outcome (Figure 5D). The further final step of the chained modeling can be performed by integrating the schema by Kravitz and Kreitzer (2012), the findings by Nazzaro et al. (2012) and our own results (Laricchiuta et al., 2012b). We suggest that by decreasing the reversal of LTP the potentiation of ECS on direct pathway might contribute to the approach behavior, prompting the animal toward the positive reinforcement (palatable food). Conversely, by increasing LTD the de-potentiation of ECS on indirect pathway might contribute to the avoidance behavior, prompting the animal toward the negative reinforcement (dark environment) (Figure 5E).

## Conclusions

Approach and avoidance behaviors are the foundation of emotional and motivational experience. These behaviors are modulated by the functioning of the network encompassing the subcortical structures implicated in the action (amygdala, dorsal striatum, cerebellum) and re-action (amygdala, hypothalamus) to salient stimuli. The nodes of this network are strongly interconnected and the final behavioral output probably depends upon the weight of the various nodes. By acting on them the endocannabinoid and dopaminergic systems increase the intensity of appetitive or defensive motivation (Häring et al., 2011; Fiorillo, 2013; Ohno-Shosaku and Kano, 2014; Piomelli, 2014). Large individual differences in endocannabinoid and dopaminergic transmission at the striatal, limbic and cortical level have been described in animals (Verheij and Cools, 2008; Yamamoto et al., 2013; Coria et al., 2014; Flagel et al., 2014) and humans (Moresco et al., 2002; Van Laere et al., 2009), as if the primitive model of response to salient stimuli is maintained as a “phylogenetic footprinting” that allows survival and adaptation.

## Author’s contributions

Daniela Laricchiuta and Laura Petrosini wrote the paper and revisited it critically for important intellectual content, approving the final version of the paper and agreeing to be accountable for all aspects of the work.

## Conflict of interest statement

The authors declare that the research was conducted in the absence of any commercial or financial relationships that could be construed as a potential conflict of interest.
